# Epidemiological characterization of pityriasis versicolor and distribution of *Malassezia* species among students in Hai Phong city, Vietnam

**DOI:** 10.18502/CMM.6.2.2838

**Published:** 2020-06

**Authors:** Bac Duy Nguyen, Hien Thi Thanh Vo, Mai Dinh Thi Thanh, Thai Van Vu, Thuy Thi Thanh Lai, Mui Thi Nguyen, Anh Thi Hong Bui, Khuong Van Trinh, Loi Ba Cao, Sang Tien Trieu, Dung Thi Kim Le, Sa Cao Hoang, Anh Tran Le, Luc Khac Nguyen, Anh Ngoc Do

**Affiliations:** 1 Department of Genetics and Cytogenetics, Institute for Military Medical Research, Military Medical University, Ha Noi, Vietnam; 2 Department of Medical Parasitology, Hai Phong University of Medicine and Pharmacy, Hai Phong, Vietnam; 3 Department of Clinical Parasitology, National Institute of Malaria Parasitology and Entomology, Ha Noi, Vietnam; 4 Department of Medical Biology and Genetics, Military Medical University, Ha Noi, Vietnam.; 5 Ha Noi University of Public Health, Ha Noi, Vietnam; 6 Department of Medical Parasitology, Military Medical University, Ha Noi, Vietnam

**Keywords:** Hai Phong city, *Malassezia*, Pityriasis versicolor, Students, Vietnam

## Abstract

**Background and Purpose:**

Pityriasis versicolor (PV) is a common fungal skin infection caused by *Malassezia* species. Previous studies have shown that the prevalence of PV is influenced by geographic factors. The aim of the current study was to find the epidemiological characteristics of PV and distribution of *Malassezia* species in the secondary school students living in Hai Phong city, Vietnam.

**Materials and Methods:**

This study was conducted on 1357 students within the age range of 10 - 16 years selected from four secondary schools in Hai Phong city. The students were screened for PV skin lesions from August 2016 to December 2017. The isolates of *Malassezia* from PV patients were analyzed by performing direct microscopy and culturing on modified Dixon agar plates, containing gentamicin, at 32oC for 7 days. In the next stage, the fungal strains obtained from patients with positive fungal cultures were identified using the CHROMagarTM *Malassezia* medium, polymerase chain reaction-restriction fragment length polymorphism techniques, and D1/D2 rDNA genome sequencing.

**Results:**

Pityriasis versicolor was diagnosed in 305 (22.48%) students and confirmed by clinical appearance and direct examination. A total of 293 (96.07%) samples grew on modified Dixon agar. With regard to demographic characteristics, 50.49% of the PV cases were female, and 57.38% of cases resided in urban areas. Furthermore, 88.52% of the subjects had the illness duration of more than 6 months. Hypopigmented and erythematous skin lesions were also observed in the research participants, with hypopigmentation being the most frequent condition (97.05%). Most of the *Malassezia* fungal strains were isolated from the back (39.56%), face (23.99%), and chest (16.51%). *Malassezia furfur* and *M. japonica* accounted for PV in 96.25% and 3.75% of the cases, respectively. Furthermore, *Malassezia furfur* was distributed in both rural and urban areas, while *M. japonica* was found only in the urban areas.

**Conclusion:**

The findings of the present study were indicative of the high prevalence of *Malassezia* yeasts, mostly *M. furfur*, among the students in Hai Phong city, Vietnam

## Introduction

The lipophilic yeasts of genus *Malassezia* are the members of the resident skin microflora in humans and other warm-blooded animals [ 1 , 2 ]. These yeasts are associated with some skin diseases, such as pityriasis versicolor (PV), seborrheic dermatitis, scalp dandruff, atopic dermatitis, and folliculitis [ 3 , 4 ]. To date, 14 species have been identified in *Malassezia* genus [ 5 , 7 ], 10 cases of which have been isolated from humans. These species include *M. dermatitis*, *M. furfur*, *M. globosa*, *M. japonica*, *M. obtusa*, *M. pachydermatis*, *M. restricta*, *M. sloofﬁae*, *M. sympodialis* and *M. yamatoensis* [ 6 , 8 ].

*Malassezia* species are a normal part of human commensal skin flora; however, they could be responsible for cutaneous diseases, mainly PV [ 6 ]. The risk factors for PV are high temperature, high relative humidity, fatty skin, corticosteroid treatment, immunodeficiency diseases, and overcrowded households [ 9 ],[ 10 ]. In addition, some studies have shown that *Malassezia* may be associated with pachydermatis fungemia, cephalic pustulosis and fungal bloodstream infections, especially in neonates [ 6 , 11 , 12 ].

*Malassezia* species can be identified by phenotypic, biochemical, and physiological techniques [ 7 ]. However, the differentiation of these species, based on these methods is difficult because some species have very similar characteristics, especially for the newly identified species [ 6 , 7 ]. Because of the limitations of morphological and biochemical methods, several molecular approaches, using different targets, have been successfully used for the species identification of *Malassezia*. Some of these approaches include polymerase chain reaction-restriction fragment length polymorphism (PCR-RFLP) based on ITS-rDNA regions and 26S genes [ 6 , 9 , 13 ], nested PCR [ 2 ], multiplex PCR [ 14 ], real-time PCR [ 7 ], and sequence analysis [ 15 ].

The secondary school students in Vietnam (10-16 years old) begin to go through puberty; therefore, they have highly active sebaceous glands. These students are at a playful age with excessive sweating. Based on the literature, *Malassezia* exists in different countries, including Vietnam [ 1 , 16 , 17 ]. Nevertheless, there are limited data on *Malassezia* infections in the secondary students in Hai Phong city, Vietnam. Hai Phong is located in the north of Vietnam with the all-year averaging temperature of 23-26oC and humidity of 80-85%. With this background in mind, the present study was conducted to determine the epidemiological characteristics of PV and distribution of *Malassezia* species in the secondary school students living in Hai Phong city, Vietnam.

## Materials and Methods

***Study population***

A total of 1,357 students within the age range of 10-16 years were selected from four secondary schools, namely Vinh Niem (Le Chan district), Lac Vien (Ngo Quyen district), Quang Hung (An Lao district), and Doan Xa (Kien Thuy district), in Hai Phong city, Vietnam, from August 2016 to December 2017. All these students were screened for PV. A questionnaire was used to record the informative data about the epidemiology of each person.

***Sampling***

The specimens were taken by scraping the lesions with a sterile blade. In case of the presence of normal subjects or insufficient scales, the samples were taken by means of sellotape. In patients with PV having more than two lesion sites, all lesions were sampled, and a record was made regarding the affected body site.

***Direct microscopy and culture***

The diagnosis of PV was based on clinical appearance and direct microscopic examination in 20% potassium hydroxide (KOH) and staining with methylene blue. To this end, the skin specimens obtained from patients with clinically suspected PV were used for direct microscopic examination with 20% KOH (RedStar Co. Ltd, Vietnam) and staining with methylene (Merk, Germany). The PV was established based on the characteristic clusters of spores with short hyphae. Skin scale sampling was continued in the patients who had positive direct microscopy, and the samples were immediately cultured on modified Dixon agar plates, containing gentamicin (at a final concentration of 25 μg/ml). 

The inoculated plates were incubated at 32°C and observed every day for a maximum of 7 days before negative results were noted. The colonies were sub-cultured on CHROMagarTM *Malassezia* medium (CHROMagar, France) to determine the co-infection rate of *Malassezia* species and distinguish between *M. furfur* and other *Malassezia* species according to the manufacturer’s instructions. If *Malassezia* species were the same in different body sites, it was considered to be a single isolate.

***Molecular identification of the isolated species of *Malassezia****

***DNA isolation***

The pure cultures of all *Malassezia* isolates were homogenized in 100 μl of sterile water (Corning, USA) and incubated with sorbitol buffer (1M sorbitol; 100 mM sodium EDTA; 14 mM β-mercaptoethanol) and 200 Lyticase enzyme units (L2524, Sigma-Aldrich Co. Ltd., Poole, UK) at 30°C for 60 min to destroy fungal cell membranes. In the next stage, the DNA of each individual isolate was extracted using the biologic QIAamp DNA Mini Kit (No. 51304, Qiagen, Hilden, Germany), according to the manufacturer's guideline. The purified DNA was preserved with distilled water at -20°C until the implementation of the PCR reaction.

***Polymerase chain reaction amplification***

The PCR products (400-550 bp) of *Malassezia*, containing ITS2 rDNA, were amplified by ITS3 (5'-GCA TCG ATG AAG AAC GCA GC-3) and ITS4 (5'- TCC TCC GCT TAT TGA TAT GC-3') primers (Integrated DNA Technologies, USA) as described previously [ 9 ]. The total PCR reaction volume of 50 μl consisted of 5 µl of the DNA solution of *Malassezia*, 25 µl Master Mix 2X (Thermo Fisher Scientiﬁc, USA), 1 μl of each primer (0.2 μM), and 18.0 μl of deionized water. The PCR reaction was performed on the Thermo Mastercycler gradient cycler (Thermo Fisher Scientiﬁc, USA) with a thermal cycle at 94oC for 5 min. This was followed by 35 cycles of 94oC for 30 sec, 53oC for 30 sec, and 72oC for 45 sec and then one cycle of 72oC for 15 min and 1 cycle of 25oC for 10 min. After amplification, the products were stored at 4oC until being used.

***Restriction fragment length polymorphism analysis***

The RFLP was performed according to the method described by Rudramurthy et al. [ 9 ] to distinguish *Malassezia* species. Digestion was performed in a final reaction volume of 16 μl, consisting of 5 μl of PCR product, 1 µl (10U) of each restriction enzyme (i.e., AluI, BanI, and MspA1I) (Thermo Fisher Scientiﬁc, USA), 1 µl of 10X Tango buffer solution, and 9 μl of deionized water (Thermo Fisher Scientiﬁc, USA). After 3 h of incubation at 37oC, the enzyme was inactivated at 65oC for 15 min. For analyzing the digestion products, 6 μl of each product in addition to 1 μl of loading dye buffer was separated by 2% agarose gel in 1X TBE buffer for about 1.5 h at 90 V. The ethidium bromide staining was the visualized by ultraviolet illumination (UVP, Canada). The size of each band was determined by a 100-bp Plus Ladder molecular weight marker (Thermo Fisher Scientiﬁc, USA).

***D1/D2 26S rDNA gene sequencing***

The D1/D2 domain region of 26S rRNA genes was amplified with the primers NL-1 (5’-GCA TAT CAA TAA GCG GAG GAA AAG-3’) and NL-4 (5’-GGT CCG TGT TTC AAG ACG G-3’) (Integrated DNA Technologies, USA). The PCR products of seven strains of *Malassezia* were sent to the Apical Scientific Sdn Bhd (Seri Kembangan 43300, Selangor, Malaysia) for purification and automatic sequencing with the same primers being used for PCR. The sequences were read on the ABI 3130 Genetic Analyzer software (SeqScape Software, version 2.1).

***Data and sequence analysis***

The SPSS statistics software (Chicago, IL, USA; version 20.0) was used for processing the data in our study. A P-value less than 0.05 was considered statistically significant. The obtained sequences were then compared to the available data in the NCBI database, using the BLAST guidelines (http://blast.ncbi.nlm.nih.gov/Blast.cgi).

***Ethical considerations***

The purpose and benefits of the study were explained to the students, as well as their parents/guardians and head teachers. The inclusion criteria were: 1) willingness to participate in the study, 2) a written informed consent, and 3) informed consent of the parents/guardians. The study protocol was approved by the Scientific and Ethical Committee of the National Institute of Malariology, Parasitology, and Entomology (Hanoi, Vietnam) in November 2015 (ethics code: 1212/QĐ-VSR). Furthermore, the study was conducted in accordance with the Declaration of Helsinki Principles.

## Results

A total of 1,357 students aged 10-16 years were chosen to be screened for PV skin lesions (Figure 2 A). The mean age at the onset of PV was 13.5 years. The research population consisted of 690 males (age range: 10-16 years) and 667 females (age range: 11-16 years). The demographic characteristics of the subjects are shown in Table 1. Based on the clinical appearance and direct microscopic examination in 20% KOH and staining with methylene (Figure 2B), a total of 305 (22.48%) patients were diagnosed with PV. There were no statistically significant differences between the two genders or between the urban and rural residents in terms of the prevalence of PV (*P=0.59* and *P=0.99*, respectively).

**Table 1:** Demographic characteristics of the research population

Demographic characteristics	Total (n = 1,357)
	n (%)
Gender	
Male	690 (50.85)
Female	667 (49.15)
Place of residence	
Rural	578 (42.59)
Urban	779 (57.41)

**Figure 1:**
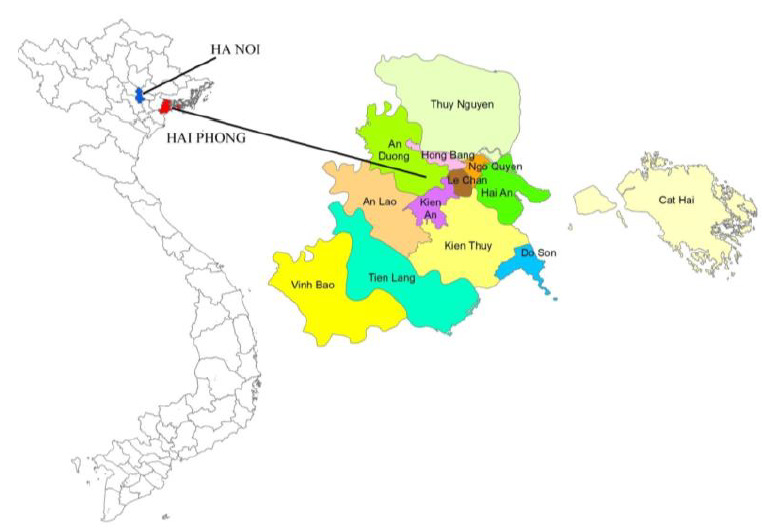
Map of Vietnam (a) Hai Phong city, north of Vietnam (b)

**Figure 2:**
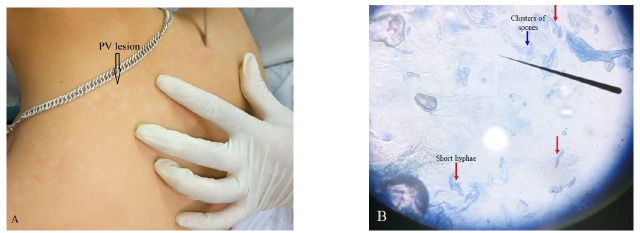
Pityriasis versicolor lesion (A) and clusters of spores with short hyphae (B) of *Malassezia* stained with methylene blue (40X)

**Figure 3:**
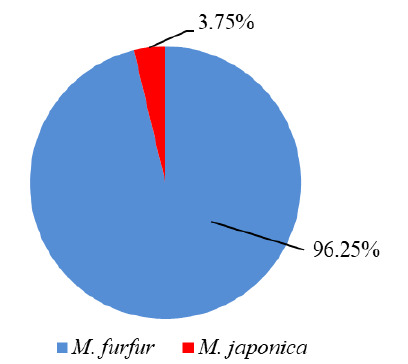
Distribution of *Malassezia* species

Based on the results, 5.25% of subjects who had PV lesion in two locations. Furthermore, out of three types of pigmentations, two types were seen in the study participants, with hypopigmentation being the most frequent one. The clinical characteristics of the subjects are presented in Table 2. Most of the specimens were collected from the lesions on the back (39.56%), face (23.99%), and chest (16.51%). Out of the 305 samples collected from PV lesions, 293 cases grew on modified Dixon agar (96.07% growth rate). Based on the results of CHROMagarTM *Malassezia* medium, PCR-RFLP, and gene sequencing methods, out of 293 *Malassezia* isolates, 282 (96.25%) and 11 (3.75%) cases were identified as *M. furfur* and *M. japonica*, respectively; however, other *Malassezia* species were not detected in the specimens (Figure 3). 

**Table 2:** Distribution of clinical characteristics of the research population

Clinical characteristics	Total [n (%)]
Gender (n = 305)	
Male	151 (49.51)
Female	154 (50.49)
Residence (n = 305)	
Rural	130 (42.62)
Urban	175 (57.38)
Duration of illness (months) (n = 305)	
< 3	28 (9.18)
3 - 6	7 (2.30)
> 6	270 (88.52)
Pruritus (n = 305)	
Yes	77 (25.25)
No	228 (74.75)
Number of affected sites (n = 305)	
1	289 (94.75)
≥ 2	16 (5.25)
Pigmentation (n = 305)	
Hypo-pigmentation	296 (97.05)
Hyper-pigmentation	0 (0)
Erythema	9 (2.95)
Combination	0 (0)
Lesion location (n = 321)	
Face	77 (23.99)
Neck	44 (13.71)
Chest	53 (16.51)
Back	127 (39.56)
Arm	7 (2.18)
Stomach	13 (4.05)

**Figure 4:**
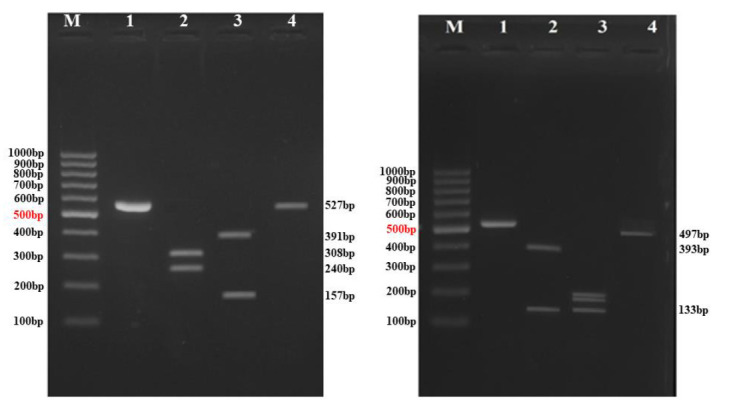
Polymerase chain reaction (PCR) product and restriction fragment length polymorphism pattern of PCR products of *M. furfur* (a) and *M. japonica* (b) after digestion with *Alu*I, *Ban*I, and *Msp*A1I enzymes; lane M) 100-bp ladder molecular weight marker, land 1 [a, b]: PCR product of approximately 550bp, and lanes 2, 3 and 4 [a, b] digestion of this product with different restriction enzymes (i.e., *Alu*I, *Ban*I and *Msp*A1I)

The prevalence of the co-colonization of the two identified *Malassezia* species was 0.34% (Table 3). These two species were discovered in both males and females. In addition, *Malassezia furfur* was found in both rural and urban areas, while *M. japonica* was only found in urban areas (data not shown). Figure 4 depicts the agarose gel electrophoresis of PCR products of the two isolates after digestion with the AluI, BanI, and MspA1I restriction enzymes. 

**Table 3:** Co-colonization of *Malassezia* species in pityriasis versicolor patients

*Malassezia* species	Number (%)
*M. furfur*	281 (96.23)
*M. japonica*	10 (3.43)
*M. furfur - M. japonica*	1 (0.34)
Total	292 (100)

The representative sequences (D1/D2 rDNA regions) of seven isolates, including three *M. furfur* isolates and four *M. japonica* isolates, were deposited in the NCBI database (GenBank, USA) with the accession numbers of MF595845.1 to MF595847.1 and MG890324.1 to MG890327.1, respectively (Table 4). 

**Table 4:** Accession number of some strains subjected to D1/D2 region sequencing and GenBank

Name of strain	Accession number	Name of species
VN-DX9C26	MF595845	*Malassezia furfur*
VN-DX7A35	MF595846	*Malassezia furfur*
VN-LV6A239	MF595847	*Malassezia furfur*
LV9D2-37-THAN	MG890324	*Malassezia japonica*
LV8C8-12-MAT	MG890325	*Malassezia japonica*
LV9D3-43-THAN	MG890326	*Malassezia japonica*
VN8B3-15-THAN	MG890327	*Malassezia japonica*

## Discussion

Pityriasis versicolor is observed more commonly among teenagers and young adults, especially in tropical and temperate regions [ 13 , 18 ]. The prevalence of PV is higher in tropical climates (nearly 30-40%), compared to that in temperate temperatures (1-4%) [ 16 ]. The relationship among disease, environment, and host factors has not been clearly described yet [ 1 ]. Moreover, there are no clear data on the pathogenesis of skin condition and the association of new *Malassezia* species with PV lesions [ 18 ]. With regard to the host, the prevalence of *Malassezia* infection depends on various factors, such as age, gender, body position, environmental, and endogenous factors [ 1 ]. Based on a body of evidence, the prevalence and distribution of *Malassezia* species depend on the identification techniques, location, and local micro-environmental variation [ 6 , 19 ]. 

In our study, 100% of the specimens were positive in the direct microscopic examination in 20% KOH and staining with methylene blue and showed clusters of yeast cells with short hyphae. The prevalence of PV was found to be higher in the secondary students (22.48%) investigated in this study, compared to the values reported for other countries, such as Iran [ 6 ] and Nigeria [ 20 ]. However, out of the 305 samples collected from PV lesions, a positive growth rate only accounted for 96.07% (n=293) of the cases. In the same vein, previous studies have shown that *Malassezia* growth rates are usually lower in the culture method than in the direct microscopic examination [ 1 , 21 ].

*Malassezia* fungi can be found in the wrinkled sites of the body. The existence of these fungi depends on some factors such as, humidity and the amount and composition of skin lipids [ 1 ]. The influence of gender on the propensity to developing PV is still unclear [ 6 ]. Our results regarding the influence of gender on PV are in line with those of the previous studies. However, other authors reported a higher incidence in women. The reasons for such differences in the rate of *Malassezia* between males and females may be attributable to the extra attention of women to their beauty and skin hygiene [ 22 , 23 ]. On the other hand, males are more involved in outdoor activities which place them at a high risk of exposure to some predisposing factors, such as high temperature and humidity [ 24 ]. 

In the currents study, the frequency of hypopigmentation lesions accounted for 97.05% of the PV lesions, which were more common than other lesions. In a study performed on 139 PV cases in Mumbai, India, Shah et al. [ 25 ] reported that 84.17%, 8.63%, and 7.19% of the PV lesions had hypopigmentation, hyperpigmentation, and both hyperpigmentation and hypopigmentation, respectively. Likewise, in a study conducted on 98 PV patients in Indonesia, as the neighboring country of Vietnam, hypopigmentation was found to be the most common lesion (64.3%) [ 16 ]. The predominance of *M. furfur* in tropical climates is probably explained by its pityriacitrin production (i.e., an ultraviolet-absorbing indole alkaloid from *M. furfur*) [ 13 , 16 ]. This agent has the ability to protect fungus against ultraviolet exposure and induce the apoptosis of human melanocytes [ 16 ] that may explain over 90% hypo-pigmentation observed in our study.

## Conclusion

As the findings indicated, the prevalence of PV was higher in the students living in Hai Phong city than in those residing in other parts of the world. In the present study, *M. furfur* and *M. japonica* were identified as the etiological agents, with *M. furfur* being more common than *M. japonica*. However, no other *Malassezia* species was detected.
